# *In vitro* evaluation of a prodrug approach for Gly-D-P18, a host defence peptide and novel anticancer agent

**DOI:** 10.1186/1753-6561-9-S1-A41

**Published:** 2015-01-14

**Authors:** A Hsu, Marc Devocelle, Joseph Ward, Stephen Keely

**Affiliations:** 1 Royal College of Surgeons in Ireland, 123 St. Stephen’s Green, Dublin 2. Ireland; 2 School of Pharmacy, Royal College of Surgeons in Ireland, 123 St. Stephen’s Green, Dublin 2. Ireland; 3 RCSI Molecular Medicine Laboratory, Beaumont Hospital, Dublin 9. Ireland

## Background

Host defence peptide (HDP) has multiple properties [1,2] potentiating it as a novel anticancer agent. However disadvantages include systemic toxicity [3]. To address this, a prodrug was developed and the aim was to assess toxicity differentials between this prodrug and its active peptide component on T84 colonic carcinoma cells. Prodrug bio activation mechanism was also assessed by use of a Cathepsin B inhibitor.

## Methods

Two peptides were provided: Gly-D-P18 and its prodrug form. The prodrug, containing a linker which will serve as substrate for a tumour associated protease, Cathepsin B, and activate the drug. T84 cell lines were cultured separately with Gly-D-P18 and its pro drug at concentrations of 1 µM and 10 µM over 24 hours. Effects were evaluated by LDH assay, Transepithelial resistance and Electrophysiological measurements. Cathepsin B inhibitor was also incubated, at concentration of 10 µM, 1 µM, 200 nM, and 4nM with pro drug on T84 cells over 24 hours and their effects assessed by transepithelial resistance and LDH measurement.

## Results

Pro drug caused a drop to 74.45% of initial resistance for 1 µM (n=5) and 22.56% for 10 µM (n=5) concentrations, in comparison to Gly-D-P18 with 52.33% (n=5) and 21.676% (n=5) respectively. Also, the use of 10 µM prodrug with Cathepsin B inhibitor at 10 µM (n=3), 1 µM (n=3), 200 nM (n=3), 4 nM (n=3) concentrations resulted in a drops to 34.12%, 26.974%, 30.009%, 25.977% of initial resistance respectively, compared to 26.804% of initial resistance from standalone prodrug (n=3) treatment. No effects were seen with regards to LDH release or chloride secretion.

## Conclusions

While the prodrug had comparatively decreased resistance drop, inconclusive results and limitations indicated need for further experimentation. In future, one could include usage of wider range of viability tests and comparisons against treatment with prodrug with uncleavable linkers as well as on healthy cells.
